# Unexpected conversion from hypothyroidism to an euthyroid state due to Graves’ disease in a patient with an ectopic thyroid

**DOI:** 10.1007/s12020-013-0117-6

**Published:** 2013-11-27

**Authors:** Ewelina Szczepanek-Parulska, Marek Ruchala, Aleksandra Hernik

**Affiliations:** Department of Endocrinology, Metabolism and Internal Medicine, University of Medical Sciences, 49 Przybyszewskiego St., 60-355 Poznan, Poland

**Keywords:** Ectopic thyroid, Graves’ disease, Follicular neoplasm

## Abstract

A 15-year-old woman was diagnosed with hypothyroidism and unilateral ectopic thyroid. Levothyroxine treatment was introduced; however, the patient was non-compliant and took the medication irregularly. When she presented to an endocrinologist at the age of 30, she had not been using levothyroxine for at least 6 months. Surprisingly, she was clinically and biochemically euthyroid. Due to decreased echogenicity on ultrasound examination, enhanced vascularization on Color Doppler examination and increased concentration of anti-TSH receptor autoantibodies, she was diagnosed with Graves’ disease. Eventually, she underwent total thyroidectomy due to diagnosis of follicular neoplasm in fine-needle aspiration biopsy of the focal lesion found in the thyroid gland. To our knowledge, our patient is the first described with ectopic thyroid, presenting a nodular variant of Graves’ disease and no signs of orbitopathy, who was initially hypothyroid and became euthyroid in the course of autoimmune thyroid disease.

**Electronic supplementary material:**

The online version of this article (doi:10.1007/s12020-013-0117-6) contains supplementary material, which is available to authorized users.

## Endocrine imaging

A 30-year-old woman was referred for fine-needle aspiration biopsy (FNAB) of a lesion located in an ectopic thyroid. At the moment the patient was born (1982), neonatal screening for congenital hypothyroidism had not yet been introduced in Poland. Hence, the data on her TSH value at birth are not available. The patient was diagnosed with hypothyroidism and thyroid ectopy at the age of 15. The ultrasound examination showed the absence of the thyroid gland in the typical localization but the scintiscan revealed an ectopic thyroid in the left submandibular region of the neck. The estimated 24-h thyroid uptake was 53 %. Her initial TSH was 22.61 μlU/ml (normal 0.27–4.20). Levothyroxine substitution was initiated at the dose of 50 μg daily and then continued with 75 μg daily. However, the patient was non-compliant and took the medication irregularly. The patient’s family history was unremarkable. Her parents did not present features of hypothyroidism. However, they were not willing to undergo further diagnostic tests. She did not have siblings or children.

**Fig. 1:**
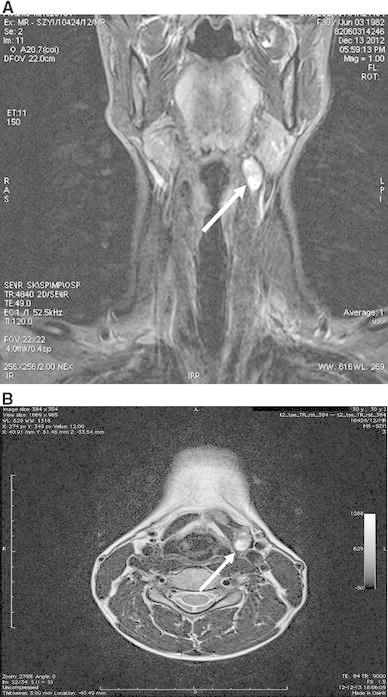
**a** T2-weighed MRI STIR (short inversion time inversion recovery) image in coronal plane. An *arrow* indicates an ectopic thyroid, **b** T2-weighed MRI STIR image in transverse plane. An *arrow* indicates a focal lesion in an ectopic thyroid

On admission, the patient was clinically and biochemically euthyroid [TSH 1.71 μIU/ml, FT_4_ 12.91 pmol/l (normal 11.5–21.0), and FT_3_ 4.76 pmol/l (normal 3.93–7.70)], free of symptoms and had not been taking levothyroxine for at least six months. Physical examination revealed a palpable painless lump in the left submandibular region of the neck. Concentration of anti-thyroid peroxidase antibodies and anti-TSH receptor autoantibodies (measured with the 2nd generation BRAHMS TRAK human RIA; Berlin, Germany) was increased; 87 IU/ml (normal < 34) and >40 IU/l (normal < 2), respectively, while anti-thyroglobulin autoantibodies were 68 IU/ml (normal 10–115). No signs of endocrine orbitopathy were noticed. The ultrasound examination revealed an absence of the orthotopic thyroid gland and the presence of an unilateral ectopic thyroid localized in the left submandibular region. The bilobed ectopic gland was 5.9 ml in volume and presented markedly decreased echogenicity as well as highly increased vascularization on color Doppler examination. Additionally, a focal lesion of 15 mm in size was observed in the left thyroid lobe. The nodule presented a normal elasticity in sonoelastographic examination. FNAB of the lesion, thyroid Tc-99 m scintiscan, and a MRI of the neck were subsequently performed (Fig. 1). A MRI confirmed the presence of an ectopic thyroid located between the thyrohyoid and sternohyoid muscle on the left with a bilobed structure, with the right lobe located anteriorly toward the thyroid cartilage on the left and the left lobe placed higher, extending to the medial wall of the left submandibular salivary gland. Cytological diagnosis of the specimen obtained during FNAB was consistent with follicular neoplasm. Hence, the patient was referred for surgical treatment. A total thyroidectomy was performed and the result of the histopathological examination revealed the presence of chronic thyroiditis and fibrosis. The focal lesion in an ectopic thyroid gland was identified as a benign hyperplasic nodule with oxyphilic metaplasia.

An ectopic thyroid is a rare thyroid developmental anomaly usually presenting with congenital hypothyroidism. Euthyroidism without levothyroxine substitution is rarely observed. Our search of the English language literature revealed only three reports of patients presenting an ectopic thyroid with no thyroid tissue in the orthotopic localization and Graves’ disease, two of them associated with orbitopathy [1–3]. To our knowledge, our patient is the first person described with an ectopic thyroid presenting with a nodular variant of Graves’ disease and no signs of orbitopathy, who was initially hypothyroid and became euthyroid presumably because of the autoimmune thyroid disease.

## Electronic supplementary material

Below is the link to the electronic supplementary material.

Supplementary material 1 (DOC 1032 kb)
