# Supplementary material for: Unexpected conversion from hypothyroidism to an euthyroid state due to Graves’ disease in a patient with an ectopic thyroid
Source: Endocrine. 2013 Nov 27;46(3):684–5. doi: 10.1007/s12020-013-0117-6 (PMC4102823; doi:10.1007/s12020-013-0117-6)
Supplement: Supplementary file 1 — Supplementary material 1 (DOC 1032 kb) [file 12020_2013_117_MOESM1_ESM.doc]

**Supplementary files**

**Figure 1.** Patient neck showing an ectopic thyroid (arrow).

**Figure 2.** Thyroid ultrasound examination. A - Color Doppler of the thyroid showing an increased blood flow. B - Thyroid nodule.

**Figure 3.** Tc-99m scintiscan of the neck. Marker indicates a sternal notch.

**Figure 1**

**
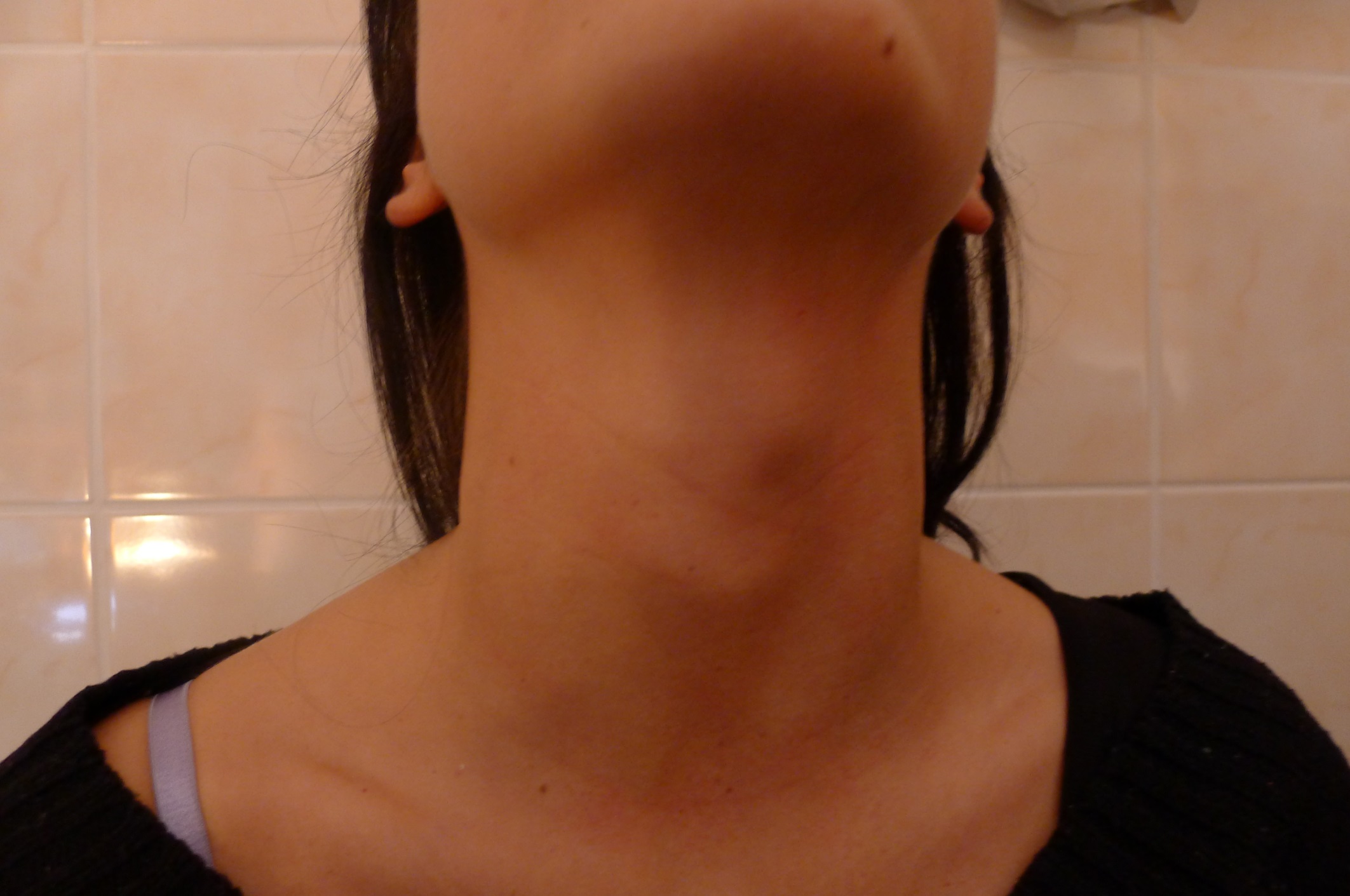
**

**Figure 2**

**Figure 2**

**A)**

**
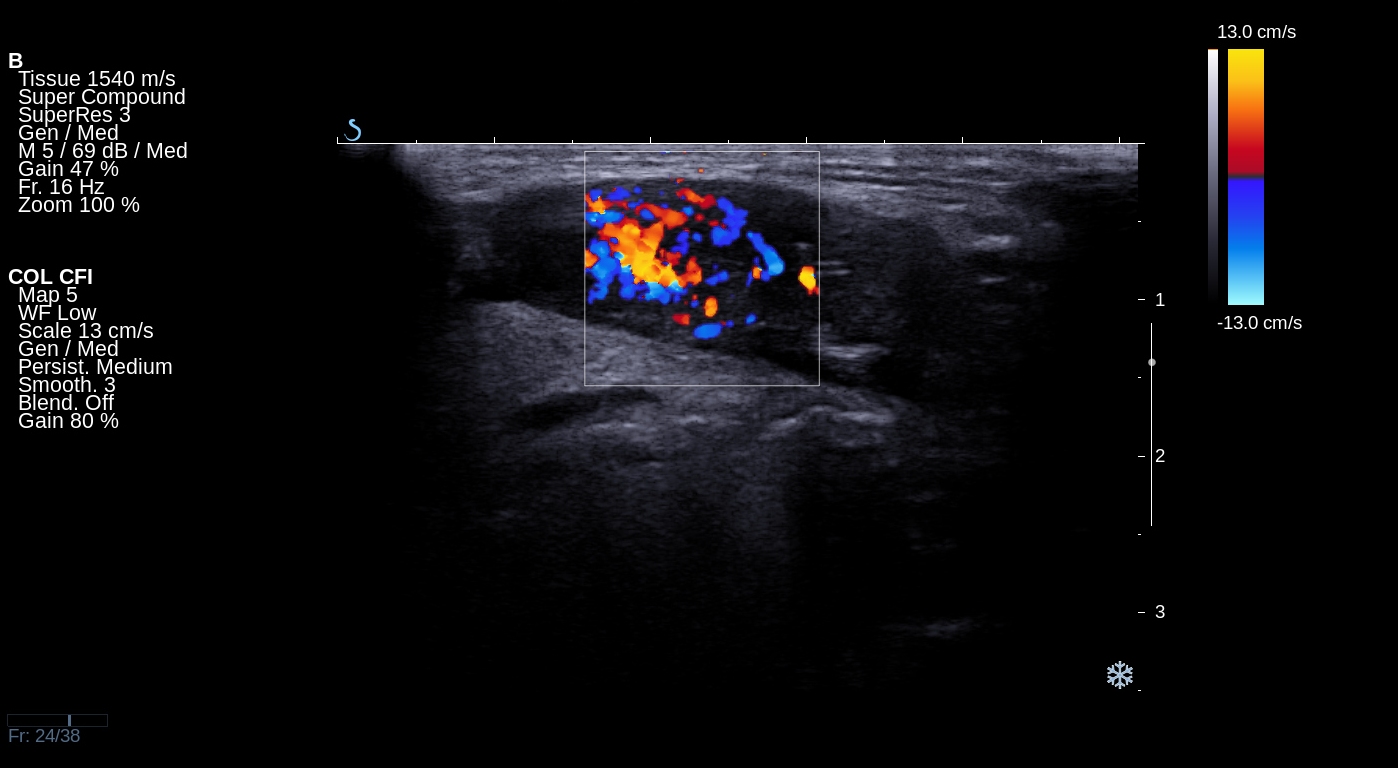
**

**B)**


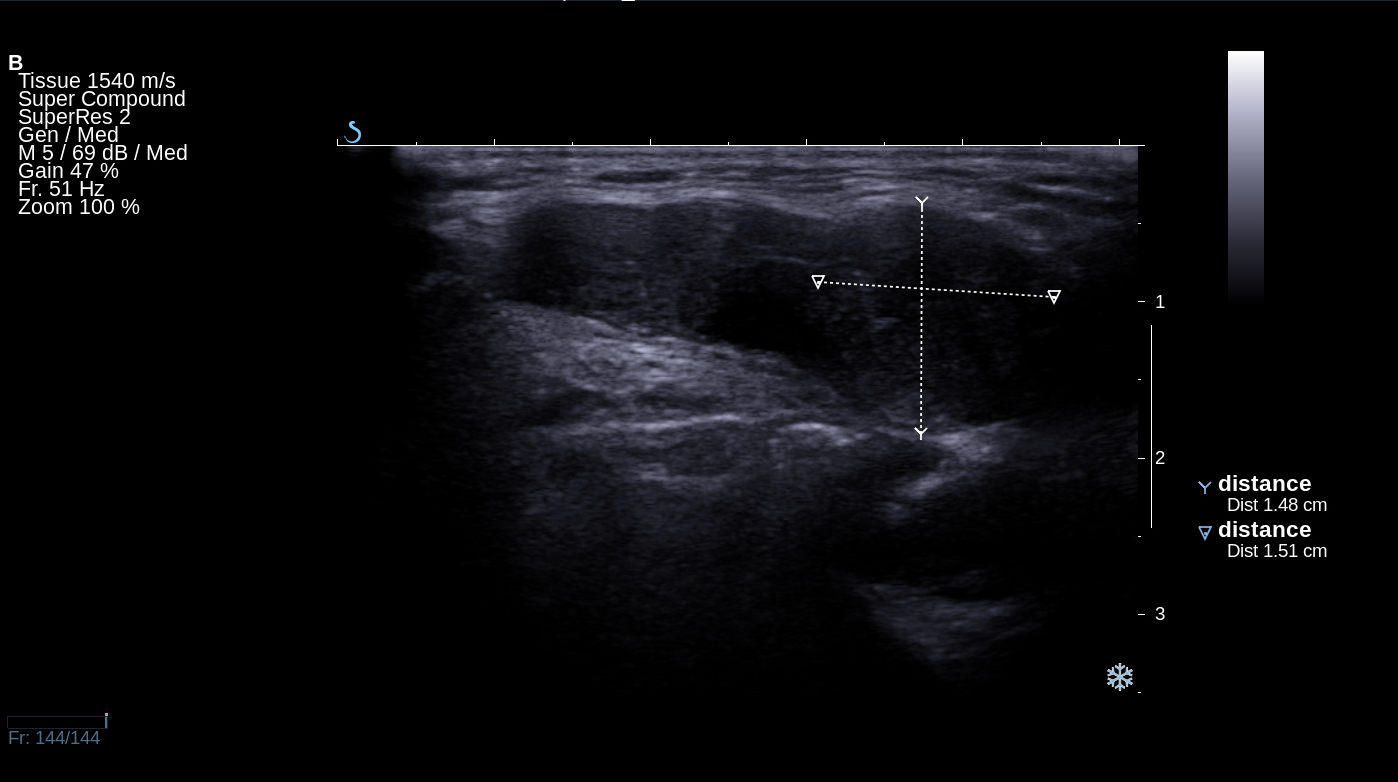


**Figure 3**

**
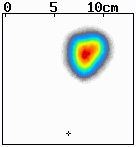
**
